# Psychedelics and sexual functioning: a mixed-methods study

**DOI:** 10.1038/s41598-023-49817-4

**Published:** 2024-02-07

**Authors:** Tommaso Barba, Hannes Kettner, Caterina Radu, Joseph M. Peill, Leor Roseman, David J. Nutt, David Erritzoe, Robin Carhart-Harris, Bruna Giribaldi

**Affiliations:** 1 Department of Medicine, Centre for Psychedelic Research, Imperial College London, London, UK; 2 Psychedelics Division, Neuroscape, Department of Neurology, University of California San Francisco, San Francisco, United States

**Keywords:** Clinical pharmacology, Human behaviour

## Abstract

Do psychedelics affect sexual functioning postacutely? Anecdotal and qualitative evidence suggests they do, but this has never been formally tested. While sexual functioning and satisfaction are generally regarded as an important aspect of human wellbeing, sexual dysfunction is a common symptom of mental health disorders. It is also a common side effect of selective serotonin reuptake inhibitors (SSRIs), a first line treatment for depression. The aim of the present paper was to investigate the post-acute effects of psychedelics on self-reported sexual functioning, combining data from two independent studies, one large and naturalistic and the other a smaller but controlled clinical trial. Naturalistic use of psychedelics was associated with improvements in several facets of sexual functioning and satisfaction, including improved pleasure and communication during sex, satisfaction with one’s partner and physical appearance. Convergent results were found in a controlled trial of psilocybin therapy versus an SSRI, escitalopram, for depression. In this trial, patients treated with psilocybin reported positive changes in sexual functioning after treatment, while patients treated with escitalopram did not. Despite focusing on different populations and settings, this is the first research study to quantitively investigate the effects of psychedelics on sexual functioning. Results imply a potential positive effect on post-acute sexual functioning and highlight the need for more research on this.

## Introduction

Between the 1950s and the 70 s, psychedelic substances such as LSD were studied in clinical settings for the treatment of mood disorders and alcohol dependence in particular^1^. In the 1960s, psychedelics became associated with the ‘hippy’ subculture, whose anti-war and sexually liberal values were encapsulated by the playful slogan “Make Love Not War”^2^. Scientific research with psychedelics was abruptly stunted by the 1971 United Nations Controlled Substances Act^1^, but it has been revived in recent decades, with several trials supporting the promise of psychedelic-therapy as a mental health intervention^3^. Psychedelics and therapeutic support are believed to act synergistically on the patient, leading to therapeutic experiences like emotional catharsis, ego dissolution, and psychological insights^4^. One area of particular promise has been psilocybin-therapy for anxiety and depressive symptoms^5–8^. In one notable study, psilocybin-therapy was found to be at least as effective as a 6-week course of the selective serotonin reuptake inhibitor (SSRI), escitalopram, at reducing depressive symptoms in major depressive disorder (MDD). Moreover, the psychedelic intervention performed significantly better than the SSRI on secondary outcomes measuring well-being, general functioning and anhedonia^7^.

Major depressive disorder is one of the leading causes of disability worldwide. It is characterised by episodes of extreme low mood, motivation, ability to feel pleasure (anhedonia), and cognitive ability^9^. Despite sexual dysfunction (SD) not being classified as a core symptom of MDD in the DSM-5 criteria, it frequently presents itself in MDD cases, reported most frequently as decreased libido, arousal difficulties and absent or delayed orgasms in both women and men^10^. SD is also a common side effect of SSRIs, reported by 40% to 65% of individuals treated with those drugs^11,12^. Highly selective SSRIs like fluoxetine, escitalopram, and citalopram are especially associated with SD^13^, impairing sexual function in both depressed subjects^13^ and healthy individuals dosed with these drugs^14–16^—likely due to downstream effects on serotoninergic and dopaminergic functioning^17^. SD is therefore a risk factor for treatment adherence and resulting relapse or recurrence of a depressive episode^10,11^.

Sexual dysfunction has also been found to be associated with lower well-being in healthy populations from both cross-sectional and longitudinal research^18–20^, which is unsurprising considering that SD is known to considerably affect quality of life, self-esteem and relationship quality^21^. Converging research indeed shows that sexual satisfaction is an important part of psychological well-being, linked to subjectively related happiness^20,22,23^, meaning in life^24^ and relationship satisfaction^19,25–27^. Consequently, lower rates of depression are reported among men and women who report to be sexually satisfied^28^. Finally, several studies have cited numerous physical health benefits of sexual activity, including, but not limited to, stronger immune system function, lower blood pressure and decreased risk of prostate cancer^29^. Sexual satisfaction thus appears to be important for a satisfying and meaningful life, both in healthy subjects, and individuals with depression.

To date, some qualitative evidence indicates that psychedelic-use may have beneficial effects on the expression and acceptance of sexual feelings and behaviours^30–35^. However, to our knowledge, no contemporary quantitative studies have assessed the impact of psychedelic-use on sexual functioning and wellbeing. Nevertheless. previous research suggests that psychedelics are capable of fostering mindfulness capacities^36,37^, enduring feelings of emotional empathy and connectedness towards others^38–40^, positive attitudes towards one’s body and lifestyle^41,42^, as well as increased curiosity and openness towards new experiences^43,44^, all of which might impact on experiences of and attitudes towards sex.

By drawing on data collected from subjects consuming psychedelic substance in naturalistic settings like attending psychedelic ceremonies, we sought to assess the impact of psychedelic-use on several facets of sexual functioning and satisfaction. We further tested the same research question in a trial of psilocybin versus 6 weeks of the SSRI escitalopram in MDD patients. The term “sexual functioning” is widely used in the sexuality literature^45^ and here is defined according to the domains of experienced pleasure, sexual satisfaction, arousal, communication of sexual desires, importance of sex, and body image. We further included two self-constructed questions conceived with the aim of investigating whether psychedelic use could change people’s perceptions of sexual intercourse beyond functioning, within the domains of increased interest in sexual exploration (below defined as "sexual openness") and spirituality. Finally, we explored possible differences in these effects between male and females in Supplementary Materials. This research question is worthwhile investigating for both clinical and basic-science implications. Clinically speaking, the propensity of SSRIs to induce sexual dysfunction can affect treatment adherence and potentially lead to a relapse or recurrence of depressive episodes. With Psilocybin-assisted therapy emerging as a promising alternative, having shown favourable results in phase 1, 2a, and 2b trials, it’s important to thoroughly assess its side effects. This can provide valuable data for patients when choosing treatment options. From a basic science viewpoint, this paper strengthens the foundation built upon qualitative findings that suggest a beneficial influence of psychedelics on sexual wellbeing. Previous research has unveiled a positive correlation between mindfulness skills, intimacy/connectedness, and sexual satisfaction^46–49^. Considering the demonstrated capacity of psychedelics to enhance mindfulness and connectedness, it becomes particularly compelling to explore their potential impact on sexual functioning.

## Results

### Participants

#### Study 1

Across the combined survey samples, a total of N = 261 participants were included in the analyses who completed baseline, 4-week and 6-month endpoint assessments. A total of 1463 participants completed baseline, 718 completed key endpoint at 4-weeks and 322 completed FU at 6 months. 61 participants completed FU but did not complete either BL or Key endpoint, therefore obtaining 322–61 = 261 participants in the present analysis. 43% of those were females and 55.6% were males sex-wise. Participants were mostly from the United States (43.8%), working full-time (63.4%) and white (90.7%). A more detailed picture of participants’ demographics can be found in Table 1.

**Table 1:** Demographic information collected at baseline for Study 1.

Total N		261
Age		39.2 ± 14.5
Sex	Female	98 (43.8%)
	Male	161 (55.6%)
	Other	2 (0.6%)
Nationality	United States	82 (43.8%)
	United Kingdom	76 (19.5%)
	Canada	14 (3.2%)
	Other countries (34 in total)	89 (26.3%)
Education	None	11 (0.7%)
	High School or equivalent (GED)	52 (7.6%)
	Associate/technical degree	7 (7.1%)
	Bachelor’s degree	82 (30.1%)
	Post-graduate (Master’s or Doctorate)	109 (20.5%)
Employment	Student	42 (5.6%)
	Working full-time	124 (63.4%)
	Working part-time	47 (14.7%)
	Retired	21 (8.9%)
	Unemployed	27 (7.3%)
Ethnicity	White	248 (95%)
	Black or African American	2 (0.76%)
	Asian	9 (3.44%)
	American Indian or Alaska native	1 (0.4%)
	Unknown/prefer not to say	1 (0.4%)/5 (1.9%)
Marital status	Cohabiting with partner	37 (14.2%)
	Married	75 (28.7%)
	Divorced	23 (8.8%)
	Separated	10 (3.8%)
	Never married	112 (43%)
	Widowed	1 (0.3%)
	Prefer not to say	3 (1.2%)
Sexual orientation	Entirely heterosexual	138 (52.8%)
	Largely heterosexual, but some homosexual desire	73 (27.9%)
	Largely heterosexual, but with considerable homosexual desire	13 (4.9%)
	Equally heterosexual and homosexual	12 (4.5%)
	Largely homosexual, but with considerable heterosexual desire	3 (1.1%)
	Largely homosexual, but with some heterosexual desire	9 (3.4%)
	Entirely homosexual	9 (3.4%)
	Other	4 (1.5%)
Substance used	Psilocybin mushrooms or truffles	166 (63.6%)
	LSD	53 (20.3%)
	Ayahuasca	22 (8.4%)
	Other (e.g., N,N-DMT, 5-MeO-DMT,…)	20 (7.7%)

#### Study 2

We used an intention-to-treat analysis for coherence with the main publication from this clinical trial^7^. 30 patients were randomised to the psilocybin group and 29 to the escitalopram group; constituting the entire sample from Ref.^7^. Of the 59 patients enrolled, 23 (39%) were on psychiatric medication, which they stopped at least 2 weeks before starting the trial; four (7%) had to discontinue psychotherapy (see^7^ for stopping criteria). In the escitalopram group, four participants stopped taking their escitalopram capsules before the end of the trial because of adverse effects attributed to the drug. In the psilocybin group, one participant smoked cannabis regularly during the trial and three participants missed the second psilocybin dosing day because of COVID-19 lockdown restrictions (2 in the psilocybin arm and 1 in the escitalopram arm). The mean age was 41 years, 20 (34%) participants were women, and 52 (85%) participants were White. Written informed consent was obtained from all patients. Sixteen patients reported having no partner either at baseline of follow-up on questions on pleasure, communication and satisfaction and thus were not included in the analyses of these questions. The remaining items and retrospective changes in sexual functioning were assessed in all 59 patients. For more information on participant recruitment and demographics, see^7^.

### Changes in sexual functioning and perceptions

#### Study 1

**Figure 1:**
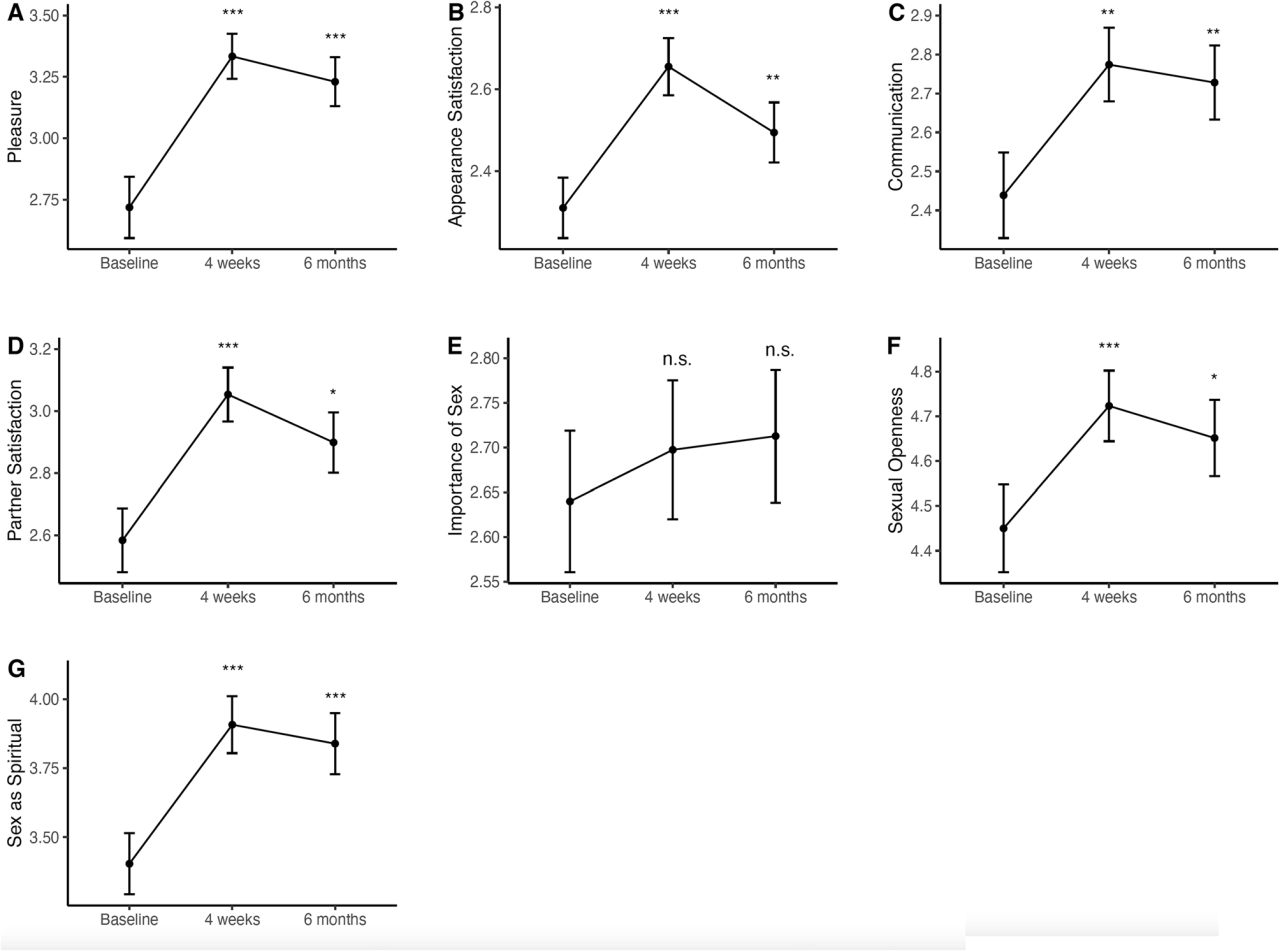
Single item analyses assessing changes in sexual functioning and satisfaction after naturalistic psychedelic use in a sample of N = 261 completers at 4 weeks and 6-month follow-up. ‘n.s’ indicates that the difference between baseline and follow-up scores is non-significant (P > 0.05). ***The difference between baseline and follow-up scores is significant, with a P < 0.0001. **The difference between baseline and follow-up scores is significant, with a P < 0.001. *The difference between baseline and follow-up scores is significant, with a P < 0.01. Error bars represent SE(M). Y-axis dimensions are scaled flexibly for better visibility of results.

Friedman rank tests (Table 2) showed statistically significant differences in the survey samples across time for all variables apart from “importance of sex” (χ^2^(2) = 1.9, p = 0.40), with the most significant changes seen for the following items: seeing sex as spiritual or sacred experience (χ^2^(2) = 35.6, p < 0.0001), satisfaction with one’s own appearance (χ^2^(2) = 30.5, p < 0.0001), satisfaction with one’s own partner (χ^2^(2) = 22.2, p < 0.0001) and experience of pleasure (χ^2^(2) = 20.9, p < 0.0001). Follow-up pairwise Wilcoxon signed-rank tests between baseline, 4-week, and 6-month endpoints showed that both 4-week and 6-month scores were elevated when compared with baseline, which was again the case for each item other than importance (Fig. 1). A detailed summary of the results can be found in Table 2.

**Table 2:** Changes in sexual functioning and satisfaction in Study 1.

Item	Baseline		4 weeks		6 months		Friedman test	
	$\overline{{\text{x}}}$	SD	$\overline{{\text{x}}}$	SD	$\overline{{\text{x}}}$	SD	*p*	Chi
Pleasure (1–5)	2.7	1.6	3.3	1.2	3.2	1.3	< 0.0001	20.9
Appearance satisfaction (0–4)	2.3	1.2	2.7	1.1	2.5	1.2	< 0.0001	30.5
Sexual communication (1–5)	2.4	1.3	2.8	1.2	2.7	1.3	0.01	9.2
Partner satisfaction (1–5)	2.6	1.4	3.1	1.1	2.9	1.3	< 0.0001	22.2
Importance (0–4)	2.6	1.3	2.7	1.3	2.7	1.2	0.40	1.9
Sexual openness (1–7)	4.5	1.6	4.7	1.3	4.7	1.4	0.007	9.8
Spiritual (1–7)	3.4	1.8	3.9	1.7	3.8	1.8	< 0.0001	35.6

Means ($\overline{{\text{x}}}$) and standard deviation (SD) are reported for each outcome at baseline (1 week before), key endpoint (4 weeks after) and follow-up (6 months after) a psychedelic experience. Significance and Chi-squared values are reported for Friedman rank sum tests.

#### Correlations with changes in well-being (study 1)

Significant Bonferroni-corrected spearman rank correlations between items of the BISF-W and the Flourishing Scale were detected for the following items: sexual communication with partner (rho = 0.25, p = 0.001), satisfaction with one’s own appearance (rho = 0.24, p = 0.0001), openness to try new things in one’s sex life (“sexual openness”) (rho = 0.22, p < 0.001), and sex as spiritual (rho = 0.17, p < 0.01), but not satisfaction with one’s partner (rho = 0.11, p = 0.16), or pleasure (rho = 0.15, p = 0.06).

#### Study 2

**Figure 2:**
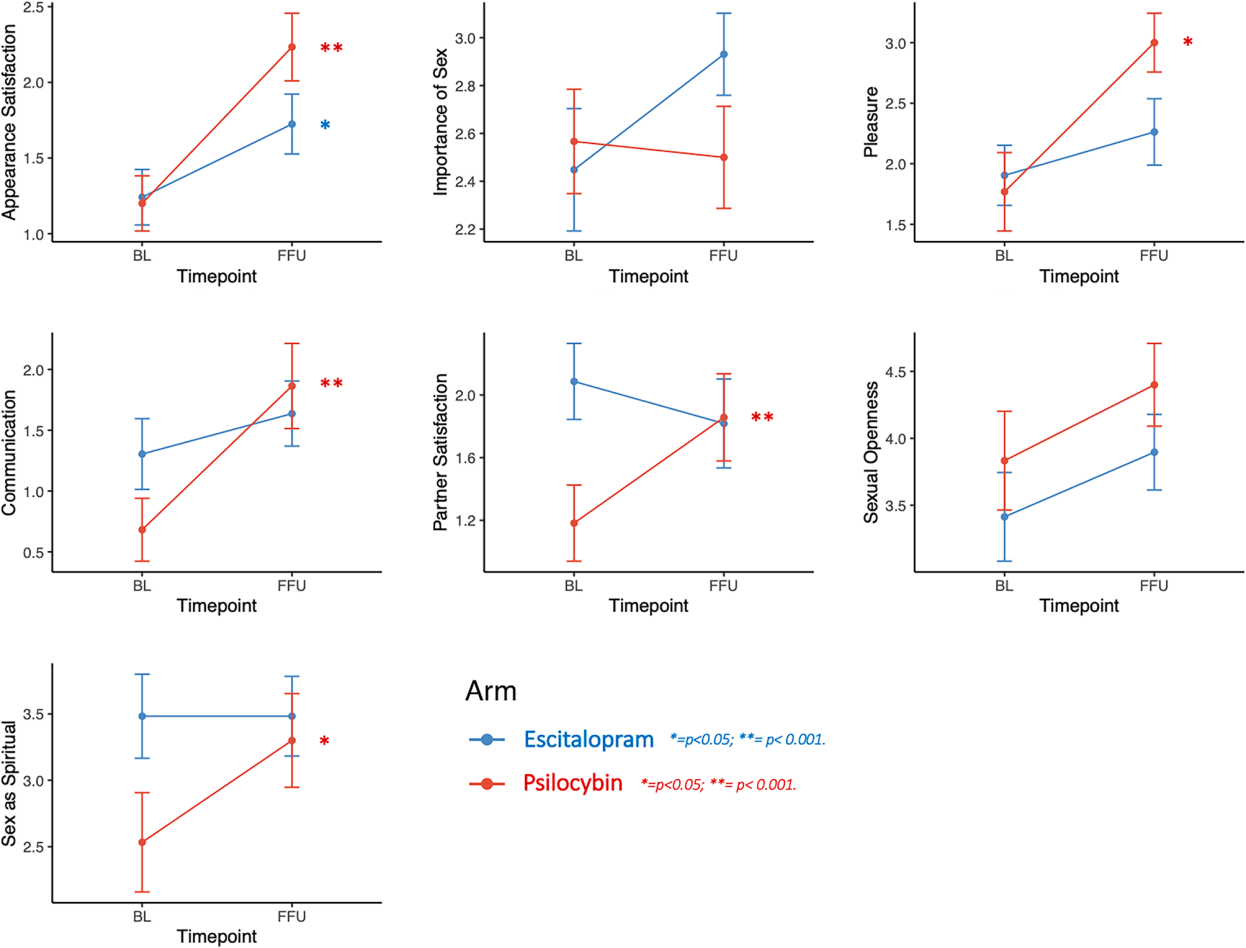
Single item analyses assessing changes in sexual functioning and satisfaction before (BL) and after (FFU) treatment with psilocybin or escitalopram in Study 2. P values indicate univariate significance in each study arm. Error bars represent SE(M). Y-axis dimensions are scaled flexibly for better visibility of results.

Across all items, except for perceived importance of sex, subjects in the psilocybin condition were more likely to experience a greater extent of positive change, indicated by the positive beta estimates (Table 3). Results of within-group post-hoc tests based on estimated marginal means derived from cumulative link models are reported in Table 3. Among the items that showed a significant interaction, post-hoc contrasts revealed psilocybin-specific improvements for the items ‘Partner satisfaction’, ‘Communication’, and ‘Sex as spiritual’. ‘Appearance satisfaction’ improved significantly in both the psilocybin and escitalopram condition, while there was a non-significant trend for perceived importance of sexuality increasing following escitalopram treatment (Fig. 2). Additionally, a significant pre-post test contrast was found in the psilocybin group for the experience of pleasure during sexual activity, despite absence of an interaction, with a change on the latent construct of 1.3 points (ΔEMM = 1.30, z = 3.10, p = 0.0019), equivalent to patients feeling pleasure 1.3 × 25% = 32.5% more frequently during sexual experiences than before treatment with psilocybin.

**Table 3:** Sexual functioning and perceptions before and after treatment for major depressive disorder with psilocybin or escitalopram in a randomized clinical trial.

Item	Psilocybin					Escitalopram					Interaction			
	Δ	SE	z	p	R	Δ	SE	z	p	R	β	SE	z	*p*
Pleasure	1.2	0.4	3.1	**0.0019**	0.89	0.4	0.3	1.1	0.27	0.27	1.4	0.9	1.7	0.121
Appearance satisfaction	1.1	0.2	5.2	** < 0.0001**	0.95	0.5	0.2	2.5	**0.014**	0.46	1.4	0.8	1.8	0.052
Communication	1.4	0.4	3.8	**0.0001**	0.69	0.4	0.4	− 1.1	0.28	− 0.20	2.1	0.9	2.2	**0.033**
Partner satisfaction	0.6	0.3	2.0	**0.042**	0.37	− 0.3	0.3	− 1.0	0.32	− 0.19	1.8	0.8	− 2.1	**0.039**
Importance	− 0.1	0.2	− 0.5	0.64	− 0.09	0.4	0.2	1.9	0.061	0.35	− 1.6	0.8	− 1.9	0.054
Sexual openness	0.6	0.3	1.8	**0.066**	0.33	0.5	0.4	1.3	0.19	0.24	0.3	0.7	0.4	0.672
Spiritual	0.9	0.3	3.1	**0.0020**	0.57	0.002	0.3	0.01	1.0	0.00	1.8	0.8	2.3	**0.021**

Longitudinal differences (Follow up—Baseline) in estimated marginal means, based on cumulative link regression models are reported for the Psilocybin and Escitalopram arm (Δ), as well as standard error (SE), z-ratio (z) and significance for each contrast. Rosenthal correlation coefficients (R) are reported as effect size (ES) estimates for within-group differences and a value of 0.00 < 0.20 indicates a very low EF, 0.20 < 0.40 low EF, 0.40 < 0.60 moderate ES, 0.60 < 0.80 strong ES, 0.80 < 1.00 very strong ES. On the right, the treatment arm vs time interaction effect is reported, including beta-coefficient, standard error, z value, and significance of the interaction.

Significant values are in bold.

**Figure 3:**
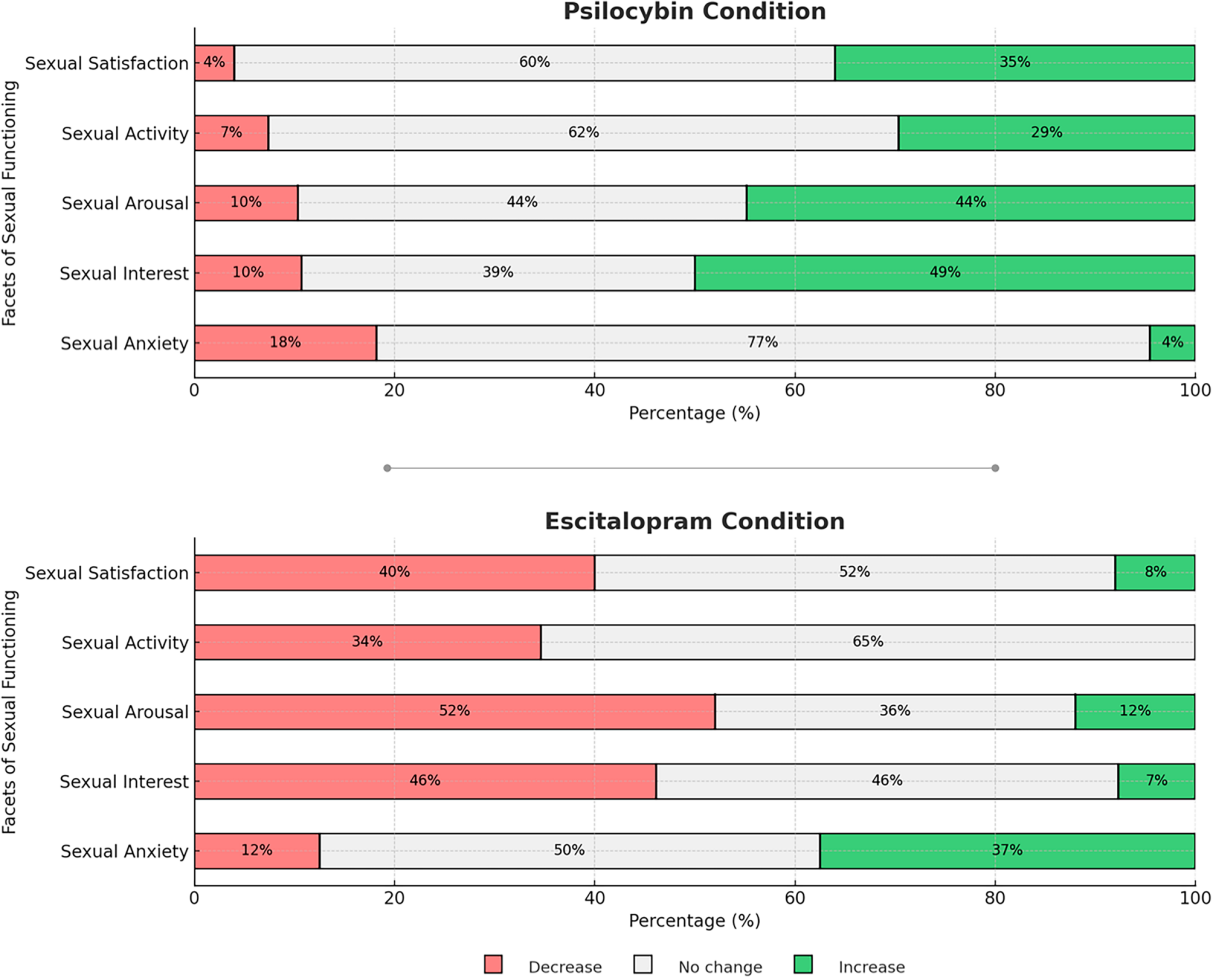
Percentage of participants who retrospectively rated decreases or increases in sexual interest, arousal, activity, satisfaction, and anxiety (reversed) after treatment with psilocybin or escitalopram at the 6 weeks follow-up of Study 2. “Increase” indicates that participants retrospectively reported an increase in the associated dimension at the end of the study compared to the beginning of it. “Decrease” indicates that participants retrospectively reported a decrease in the associated dimension at the end of the study compared to the beginning of it.

Significant differences between Escitalopram and Psilocybin’s effects on sexual functioning were identified using retrospective BISF-W item 13, which was divided into changes in sexual interest, arousal, activity, satisfaction, and anxiety. Mann Whitney-U tests showed that patients receiving psilocybin were significantly more likely than those who received escitalopram to report higher, rather than lower levels of interest (p = 0.0002), arousal (p = 0.0004), activity (p = 0.0007), and satisfaction (p = 0.0006). In each case, mean values reported by patients receiving psilocybin reflected a ‘higher level’ at 4 weeks compared with baseline, while those in the escitalopram group on average reported a ‘lower level’ compared with baseline (Fig. 3). This pattern was reversed for sexual anxiety, which was increased in those receiving escitalopram, and reduced in those receiving psilocybin, although this difference only reached significance before correction for multiple comparisons (p = 0.028; Table 4).

**Table 4:** Retrospective changes in sexual functioning after treatment with psilocybin or escitalopram.

Retrospective changes in sexual functioning (BISF-W item 13)									
Item	Psilocybin			Escitalopram			U	*p*	*r*
	M	$\overline{{\text{x}}}$	SD	M	$\overline{{\text{x}}}$	SD			
Interest	0.5	0.4	1.1	0.0	− 0.8	0.9	159	**0.0002**	− 0.49
Arousal	0.0	0.3	1.2	− 2.0	− 0.9	1.2	− 7.6	**0.0004**	− 0.46
Activity	0.0	0.1	1.4	0.0	− 0.7	1.3	− 5.0	**0.0007**	− 0.45
Satisfaction	0.0	0.4	1.3	0.0	− 0.7	1.3	− 3.2	**0.0006**	− 0.45
Anxiety	0.0	− 0.3	1.2	0.0	0.2	1.2	− 0.5	**0.028**	0.29
Composite score	1.0	1.5	1.5	− 4.0	− 3.6	1.3	− 3.0	**0.0004**	− 0.47

Median (M), Means ($\overline{{\text{x}}}$) and standard deviation (SD) are reported for each item reported 6 weeks after treatment with psilocybin or escitalopram. Significance and effect sizes (r) are reported for Mann Whitney U tests. r ≥ 0.3 was defined as a small, r ≤ 0.5 medium and r > 0.5 as a large effect. Responses were given on a scale from ‘ − 1: Lower level’ to ‘ + 1: Higher level’ since start treatment.

Significance values in bold survive Bonferroni-correction for multiple comparisons.

**Figure 4:**
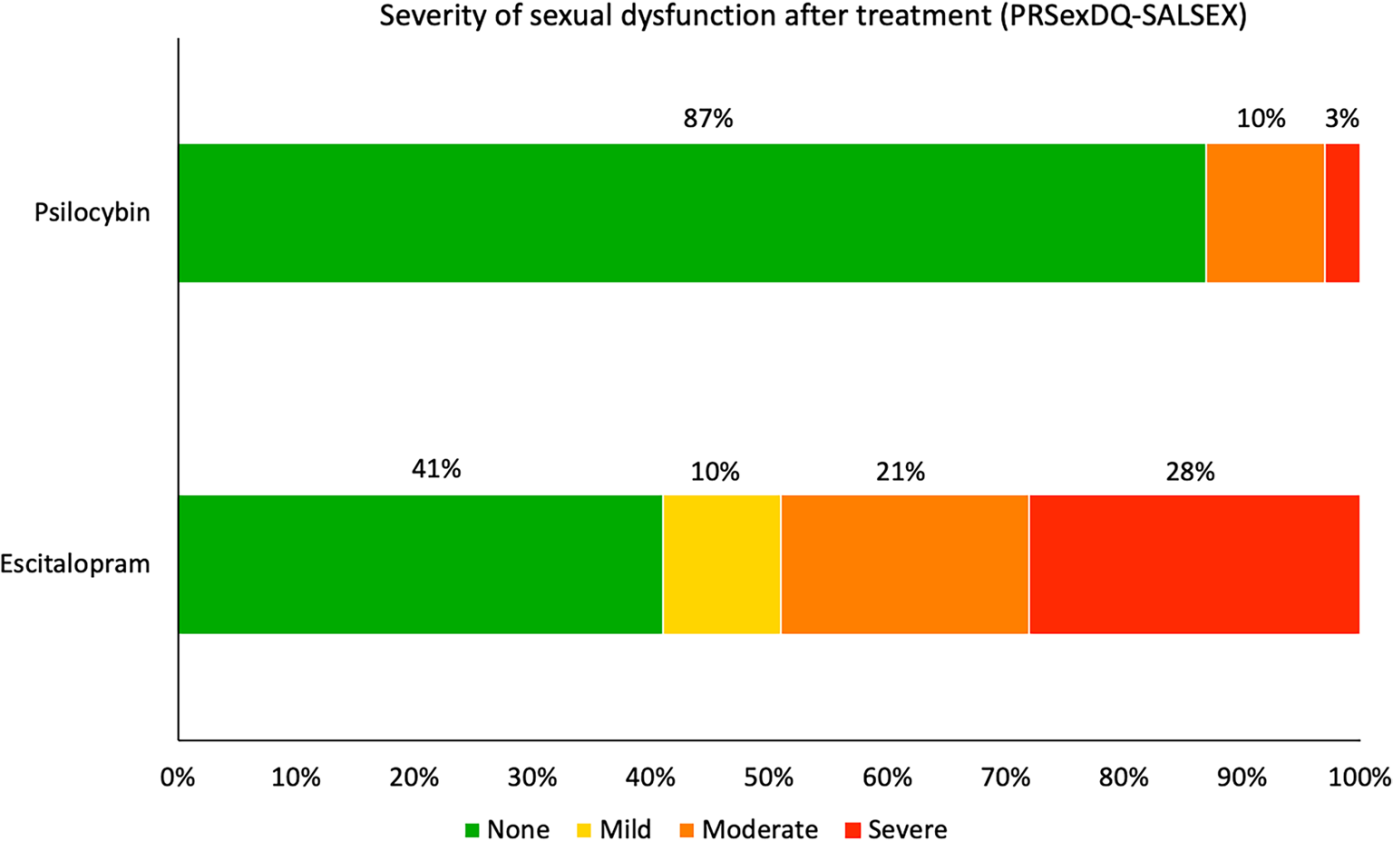
Percentage of participants who reported different degrees of sexual dysfunction after treatment with escitalopram or psilocybin at the primary endpoint of Study 2. Sexual dysfunction includes loss of libido, delayed or lack of orgasm or ejaculation, erectile dysfunction in men/vaginal lubrication dysfunction in women and patient’s tolerance of it.

Regarding sexual dysfunction (PRSexDQ-SALSEX), at the 6-week post-treatment endpoint, in the escitalopram condition 8 patients were classified as “severe”, 6 as “moderate”, 3 as “mild” and 12 as “none”. At the same endpoint, in the psilocybin condition, 1 patient classified as “severe”, 3 as “moderate” and 26 as “none” (Fig. 4). A Mann Whitney U test (U = 255.5, p = 0.001, MD = 0.98) showed that patients in the escitalopram condition were significantly more likely to have higher levels of SD severity (M = 1.3, SD = 1.3) than patients in the psilocybin condition (M = 0.3, SD = 0.8). A previous study report on this trial only reported median values when calculating PRSexDQ-SALSEX scores, but the present paper has reported the number cases pertaining to each category, which exposed the robustly significant difference between the two conditions.

#### Correlation with changes in depressive symptoms (study 2)

Collapsing the psilocybin and escitalopram groups into one, retrospectively rated changes in several aspects of sexual functioning were correlated with before-vs-after changes in depressive symptoms. Spearman rank correlations identified the strongest correlations for changes in depression and changes in both sexual arousal (Spearman’s rho = 0.38, p < 0.01) and sexual interest (Spearman’s rho = 0.36, p < 0.01), such that bigger changes in depression resulted in higher improvements in sexual arousal/interest. Correlations between changes in depressive symptoms and sexual satisfaction did not survive multiple comparison correction (Spearman’s rho = 0.31, p = 0.03) and correlations with changes in sexual activity (Spearman’s rho = 0.23, p = 0.09) and sexual anxiety change (Spearman’s rho = 0.22, p = 0.13) also did not reach significance.

## Discussion

The current study sought to examine the impact of psychedelic use on sexual functioning and satisfaction across two distinct studies and populations: one group used psychedelics for recreational and well-being purposes, while the other consisted of depressed patients. One study adopted a naturalistic observational survey approach, while the other was a controlled clinical trial. Notably, both studies and populations reported enhanced sexual functioning and satisfaction following psychedelic use.

Participants in the former study showed significant improvements in their communication with their partners, increased frequency of experiencing pleasure during sex, as well as increased satisfaction with their partners and their own physical appearance following the psychedelic experience. They also appeared to be more open to trying new things in their sex life and were more likely to perceive sex as a spiritual or sacred experience post-use. These changes were significant both 4 weeks and 6 months after the experience. However, this cohort did not report experiencing changes in the overall importance attributed to sex. Exploratory analyses aimed at investigating possible differences in these effects between males and females found no evidence of such differences, except for partner satisfaction at 6-months where we found a return of partner satisfaction levels back to baseline in female but not male participants (Supplementary Materials). Several of these changes significantly correlated with post-psychedelic changes in well-being, consistent with previous research indicating a positive association between sexual functioning and general psychological well-being^20,22,23^. Given the inherent limitations of survey studies, such as the lack of a control condition, the inclusion of individuals already particularly interested in psychedelics and the lack of control of the circumstances of psychedelic exposure, we aimed to replicate these results in controlled settings, despite focusing on a different population. Consistent with the effects reported in the naturalistic study, individuals with depression treated with psilocybin-therapy in a controlled trial setting showed improvements from baseline to post-treatment in communicating with their partners, experiencing greater sexual pleasure during sex, being more satisfied with their partner and their own appearance, and being more likely to perceive sex as a spiritual experience. Conversely, in the same trial, patients treated with a 6-week course of the SSRI escitalopram, and the same amount of therapy, only reported increased satisfaction with their appearance and no positive changes in any other domain. Furthermore, patients treated with psilocybin were more likely to report increased sexual interest, activity, arousal, and satisfaction at the 6-week endpoint than patients treated with escitalopram, who on average, reported a worsening in the same domains. Similarly, anxiety linked to sexual activity decreased for patients in the psilocybin condition and increased for those treated with escitalopram. Across both groups, changes in sexual arousal and interest were moderately correlated with changes in depressive symptoms, while changes in the other domains appeared to be somewhat independent from changes in depression. With regard to sexual dysfunction, patients treated with escitalopram were more likely to retrospectively report higher levels of sexual dysfunction after treatment compared with the individuals treated with psilocybin. These observations are consistent with recent findings from the same trial that explicit symptoms of depression related to SD (i.e., Hamilton Rating Scale for Depression-17 Libido^50^, Beck Depression Inventory-Reduced Sexual Interest^51^), as well as amotivation, anhedonia and energy levels were among the most differentially responsive to psilocybin versus escitalopram^52^. The results constitute the first empirical evidence that psychedelics might exert beneficial effects on sexual functioning and sexual wellbeing after acute use of the drug itself, consistent with previous qualitative reports indicating such an effect^30–35^. Future research to replicate and further investigate these findings is thus highly encouraged.

While we previously found that both escitalopram and psilocybin were equally effective in reducing depressive symptoms when assessed with the primary outcome of the study^7^, differences in their impact on sexual functioning and dysfunction could be explained by their differing mechanism of action in treating MDD (see^7,53^ for full discussion). It is generally thought that the pharmacological mechanisms for SSRIs-induced sexual dysfunction are intrinsically linked with their hypothesised antidepressant mechanism. By selectively inhibiting serotonin reuptake in the central nervous system (CNS), SSRIs elevate synaptic serotonin concentrations consequently increasing post-synaptic serotonin activity^11^. Generally, an increase in serotoninergic functioning appears to negatively impact on sexual functioning—perhaps as a consequence of a negative downstream effect on the production of dopamine, testosterone, acetylcholine and nitric oxide which are crucial for libido, sexual arousal and achieving orgasm in both men and women^10^. Additionally, it is also plausible that the emotional blunting sometimes induced by SSRIs might also be linked with diminished sexual functioning^54,55^. Accordingly, as previously reported in the main publication from this trial, the percentage of patients reporting emotional blunting (assessed with the Laukes Emotional Intensity Scale) and a self-constructed “Post-Treatment Changes Scale” (PTCS) at the 6-week endpoint was higher in individuals treated with escitalopram compared with psilocybin^7^. While some research suggest that the prevalence of SSRI sexual side effects may be overestimated due to a priori deterioration of sexual functioning in MDD^10^ several RCTs indicate that escitalopram^14^ and other SSRIs^15,16^ do indeed induce SD—including in healthy individuals. Such results support the view that SSRIs have a detrimental effect on sexual function beyond their impact on depression. This is clinically concerning as sexual functioning bears relevance to two core facets of depression, namely anhedonia and amotivation^56^. The occurrence of SD as a side effect of SSRIs can lead to a dilemma for both patients and clinicians. On one hand, these treatments are necessary for managing depressive symptoms, but on the other hand, they can exacerbate SD, thereby further impacting the patient’s quality of life and potentially affecting treatment adherence. Moreover, SD can contribute to the persistence or worsening of depressive symptoms, creating a vicious cycle that is difficult to break^10^. Despite the high prevalence and significant impact of SD, it is often underassessed and undertreated in mental health care settings. This oversight may be due to a variety of factors, including lack of awareness among clinicians, discomfort discussing sexual issues, or the assumption that SD is an inevitable consequence of depression or its treatment^57^. While most cases of SD associated with SSRI use tend to resolve shortly after discontinuing the medication, a minority of patients may experience enduring dysfunction, referred to as post-SSRI sexual dysfunction (PSSD^58^). PSSD is characterized by persistent symptoms such as genital anesthesia, erectile dysfunction, and pleasureless orgasm. The underlying causes of PSSD remain largely unknown, however it is acknowledged as a rare side effect associated with SSRI use^58^. Psilocybin also exerts its acute effects by acting on the serotoninergic system, but via direct agonism at serotonin 2A receptors (5-HT2AR^3^). Despite limited research on the effects of 5-HT2AR agonists on sexual activity, animal studies have indicated that 5-HT2AR agonism contributes to the inhibition of sexual activity in male rats^59,60^ while having a positive effect in females^61^. Antidepressant drugs that possess 5-HT2AR antagonist activity, such as mirtazapine and nefazodone, generally have a positive effect on SD^62^. Therefore, some have proposed that activity at 5-HT2A receptors has suppressing effects on sexual functioning in humans^10^. Nevertheless, anecdotal reports of increased sexual pleasure and intense sexual feelings under psychedelics^32,33,63^ contradict this. Clearly, more research is needed to understand the acute effects of psilocybin and other psychedelics on sexual functioning. However, it is important to note that our present study assess post-acute effects of psychedelic-use or psychedelic-therapy on sexual functioning and *not* acute effects; thus, our results should not be confused with ‘drug-sex’ or ‘chem-sex’. As such, the acute (e.g., pharmacological) effects of psilocybin on sexual functioning is not be centrally relevant here; rather, our focus has been on longer-term changes post psychedelic-use or psilocybin-therapy.

Despite not being able to directly test these hypotheses, we speculate that the results obtained from both studies might be explained by the capacity of classic psychedelics (and relatedly psilocybin-assisted therapy) to foster long-term improvements in mindfulness capacities and connectedness with significant others^37,64^, consequently impacting sexual satisfaction. Qualitative and quantitative research shows that psychedelic-use can foster non-judgement and non-reactivity^37,64^, an ability to articulate momentary experience^36,65^ and an openness to new experiences^43,44,66^. Furthermore, psychedelics appear to promote durable feelings of connection towards self and others^63,67^, increased willingness to accept and let go of one’s emotions, and decreased ruminative thinking^68^. In tandem, work by Keinplatz et al.^69^ identified eight major components that contribute to an optimal sexual experience: being present, connection, deep sexual and erotic intimacy, extraordinary communication, interpersonal risk-taking and exploration, authenticity, vulnerability, and transcendence. Subsequent research evidenced the importance of maintaining a mindful^70,71^ and open^72^ state of mind for attaining a satisfactory sexual performance. Moreover, it has been shown that increasing trait mindfulness in both women and men improved SD, including arousal/interest disorders^46,47,73–75^. Cross-sectional, longitudinal, and experimental studies also indicate that experiencing emotional connection and intimacy with one’s partner can maintain sexual desire and activity in relationships of longer duration^48,49,76^ and that a type of sexual activity understood as shared and mutual by both partners can be conductive of a better couple’s mental health^28^. Additionally, evidence from neuroimaging research^77^ previously found that female Hypoactive Sexual Desire Disorder’ (HSDD) was linked with higher levels of activity in brain regions involved in self-referential functions, such as the medial prefrontal cortex and the posterior cingulate cortex. It was suggested that HSDD might be the result of excessive cognitive activity directed toward oneself—i.e., self-consciousness, rather than naturally attending to sensory aspects of the sexual experience. Disruption of cortical activity in brain regions involved in self-referential processing has been found to be a somewhat consistent marker of the action of psychedelics^78^. By combining the results from these fields of research, it thus appears plausible that psychedelic-use, or more cautiously, psychedelic-therapy, could have a positive effect on traits associated with more embodied and satisfactory sexual experiences, freer from cognitive interferences, aversions, anxieties and demands. Additionally, we speculate that an effect of psilocybin therapy on attachment styles might have also contributed to the observed results, despite this was not directly investigated. Depression has been previously demonstrated to be linked with attachment insecurity^79^ and anxious and avoidant attachment styles have been both shown to be linked with decreased sexual satisfaction in the general population^80,81^. Psilocybin-therapy has been shown to improve attachment insecurity 3 months post-intervention^82^. Thus, the formation of a more secure attachment could have also contributed to improving sexual satisfaction. Future research should investigate this matter.

Interestingly, it was also found that participants reported perceiving sex as a more spiritual or sacred experience after psychedelic use. The rationale behind investigating this research question stems from our prior discovery that psychedelic use can amplify spiritual beliefs and attitudes towards life^83^. We are thus wanted to explore whether this increased spirituality translates into the domain of sexual experiences. While an allegiance to a religious belief system has been found to be associated with fewer life partners and lower rates of premarital and extramarital sex^84^, the link with spirituality, typically involves a ‘self-transcendent’ perspective, is less clear. Previous research indicates that ascribing spiritual or transcendent qualities to sexual intercourse is linked with increased sexual satisfaction^69,85^. However, conflicting research indicated that perceiving sex as more spiritual is not inherently positive, as spirituality has been found to be positively associated with a higher frequency of sex without a condom in women, suggesting that it might be a factor for risky sexual behaviour^84^. Additionally, participants from the survey sample appeared to be more willing to try new things in their sexual life, an effect that might be explained by increased openness to experience after psychedelic use^43,44,66^. More research investigating the links between sexual attitudes and behaviours, spirituality and psychedelic use is needed to better understand the complex relationships between these factors.

### Limitations

The findings of the present study should be considered in the context of its limitations.

Analyses in this study were conducted based on individual items of the BISF-W^86^, a previously validated measure. Given our mixed-gender sample, we chose items relevant to both sexes, focusing on domains like pleasure, communication, partner satisfaction, sex importance, and body image satisfaction. To reduce participant burden amidst multiple measures, we didn’t use the full scale. While we employed suitable statistical methods for ordinal data, future studies should use comprehensive, validated scales. We also introduced unvalidated items on viewing sex as a spiritual experience and sexual openness, without defining terms like “spiritual” and “new things”. For these reasons, caution is advised before interpreting these specific results.

Additionally, there are several distinct features and limitations to the observational study design employed in our investigation. Study 1 lacks of experimental control, potential biases towards psychedelic drugs due to opportunity sampling, demographic and other biases related to sampling and attrition issues, and reliance on subjective reporting of drug dosages. Importantly, without experimental control, we cannot establish causality or control for potential confounding factors. On the other hand, Study 2, based on RCT data, provides evidence with the experimental control that Study 1 lacks. RCTs, including ours, offer controlled settings to evaluate specific interventions, often seen as valuable in the research community for treatment evaluation. However, it is important to note that Study 1 and Study 2 cater to different contexts and realities. Study 1 assesses community-dwelling individuals, most of whom are presumably healthy and use psychedelics for recreational and wellbeing related purposes. In contrast, Study 2 evaluates the effects of psilocybin on depressed patients in a clinical setting. While the studies address different questions and settings, by presenting both observational and RCT data, our intention was to provide a broader perspective on the effects of psychedelics on sexual functioning and wellbeing. These two distinct study designs offer complementary insights into the topic, each from a different vantage point. While this approach possesses inherent limitations, our aim was to provide readers with a richer understanding by juxtaposing these two different perspectives, despite focusing on different populations and settings. Being this the first quantitative investigation on the effects of psychedelics on sexual functioning/wellbeing, we strongly encourage further research on the topic in order to overcome the current limitations.

Furthermore, future research on the effects of psychedelics on sexual functioning should consider including dyadic assessments, i.e., where the partner of the primary participant is involved, and questions that pertain to the social and cultural context of use, e.g., whether the substance was taken together with one’s partner. Relatedly, we do not know if participants engaged in sexual activities in while using psychedelics in Study 1, which could have implications for how they perceive its impact on their sexuality. However, it’s important to note that most participants in Study 1 consumed psychedelics in ceremonial settings, where sexual intercourse is strongly discouraged or even prohibited, even between romantic partners^87^. Partners are typically asked to maintain distance during these ceremonies. Nevertheless, we cannot exclude the possibility that some participants from Ref.^88^, consuming psychedelics in personal settings, engaged in such activities.

Study participants from the survey study sample and the RCT were predominantly white, sexually straight, employed and well-educated, limiting generalizability. Similar demographic data have been found in other psychedelic research studies^89^. Such consistency may imply that these demographics are reflective of the broader psychedelic-using population; however, they are not necessarily reflective of broader populations per se. Recent research has indicated that ethnoracial background moderates the health impact of psychedelic-use^90^. It is important therefore that future studies test the replicability of the present findings in more sociodemographically diverse samples. Moreover, both treatment groups benefited from extensive psychological support, with an approach inspired by the Acceptance and Commitment Therapy model^91^. Given that this model emphasizes enhancing acceptance and minimizing the suppression of challenging emotions, it might be possible that the therapeutic support acted synergistically with psilocybin to promote positive effects on sexual wellbeing. Future research should better investigate this matter, especially considering the link between sexual shame and sexual dysfunction.

The present results pertaining to escitalopram’s effects on sexual functioning cannot be generalised to other existing antidepressants, as existing research indicates that there are approved antidepressant medications on the market that do not induce SD at such high rates as SSRIs^14^. These medications have been previously advised for patients suffering from SSRI-induced SD. A further limitation of study 2 might be related to the confounding factor of antidepressant withdrawal, as the observed improvements in sexual function in the psilocybin arm could be attributed to the suspension of all antidepressants in the weeks preceding the administration of psilocybin. While only 11 out of 30 patients from the psilocybin group discontinued antidepressant medications before starting the study^7^, this could have impacted the results.

Lastly, there have been reports of sexually abusive behaviour in the context of psychedelic ceremonies and therapy^87,92^. While these dynamics are not unique to psychedelic therapies^93^, the addition of powerful mind-altering compounds in the equation requires the employment of additional caution, prevention and mitigation strategies. Relatedly, the use of psychedelic or empathogenic compounds in romantic contexts might also create complex relationship dynamics such as promoting feelings of attachment to an ordinarily undesired or abusive partner, sexual activities done under drug influence that are later regretted, or wrongly perceiving another individual as romantically or sexually interested or engaged—an issue that extends to other psychoactive drugs such as alcohol. As policies around psychedelic use evolve, it’s imperative to define clear ethical standards and professional guidelines to prevent abuse and ensure accountability. Educating individuals about potential risks and encouraging vigilance can further reduce harm and foster a safer environment for all involved.

### Conclusions

The present study contributes some first preliminary evidence that both the naturalistic and controlled therapeutic use of psychedelic drugs might foster an improvement in several facets of sexual functioning and satisfaction, including experienced pleasure, sexual satisfaction, communication of sexual desires and body image. Moreover, the present study specifically highlights that psilocybin therapy for MDD might be linked with improvements in sexual functioning. On the other hand, escitalopram—a commonly used SSRI—seemed to negatively impact sexual functioning, despite both treatments inducing similar reductions in depressive symptoms. These findings highlight the need for further research utilizing more comprehensive and validated measures to fully understand the effects of psychedelics on sexual functioning. However, the preliminary results do suggest that psychedelics may be a useful tool for disorders that impact sexual functioning.

## Methods

### Design

#### Study 1

**Figure 5:**
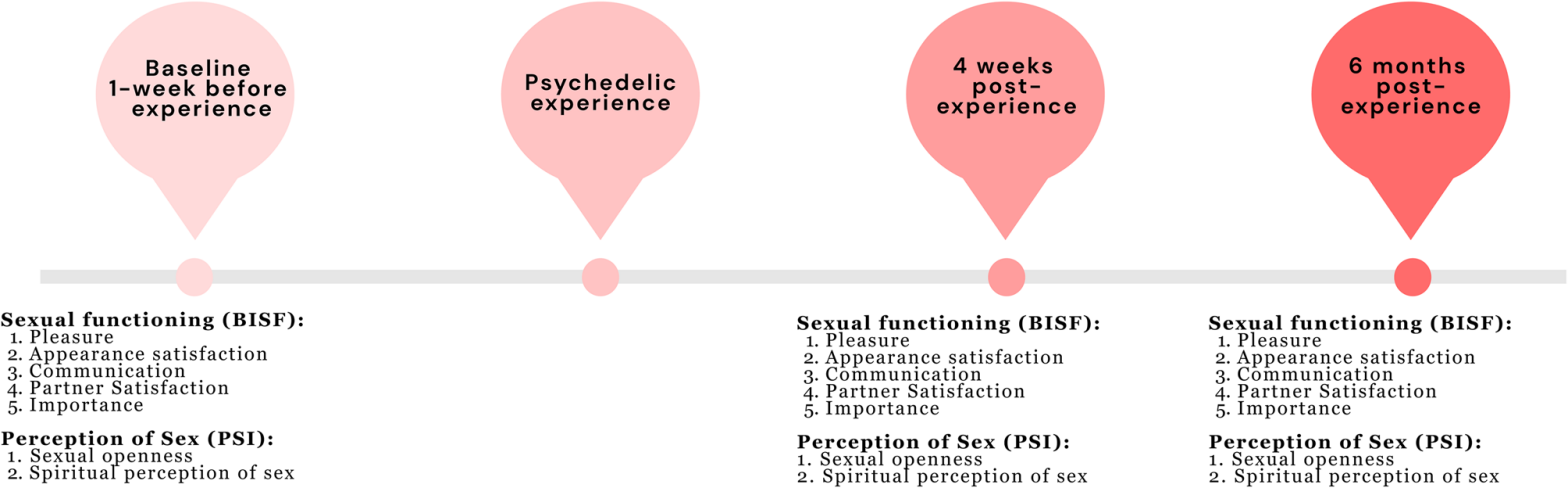
Overview of Study 1 with the included items assessing sexual functioning and perceptions of sex at the relevant timepoints.

The present study combined datasets from two large prospective online survey studies investigating the impact of psychedelics consumed in personal and ceremonial settings in the real world. All studies collected data using the bespoke online software platform www.psychedelicsurvey.com and the online platform Alchemer. The first cohort survey study^88^ recruited participants who were already planning to consume psychedelics in the near future, outside of a research or organised ceremonial setting. The second dataset comes from a survey study targeted towards individuals planning to attend an organised ‘ceremony’ entailing the consumption of a classic psychedelic substance (psilocybin/magic mushrooms/truffles, ayahuasca, DMT, San Pedro, LSD/1P‐LSD), e.g., in a psychedelic retreat or other form of guided psychedelic experience^38^. Both studies received a favourable opinion from the Imperial College Research Ethics Committee and were sponsored by the Imperial Joint Research and Compliance Office, and all participants were 18+ years old, recruited online and provided informed consent. In all three survey studies, participants were prompted to select the date of their future psychedelic experience, and questionnaires were automatically sent to them 1 week before the experience (baseline), and 4 weeks and 6 months after the experience. All methods were carried out by respecting/adhering to relevant guidelines and regulations. An overview of study 1 timepoints can be found in Fig. 5. Extensive information about the design of these two prospective online surveys can be found in Refs.^38,88^. CONSORT diagram for Study 1 can be found in Supplementary Materials.

#### Study 2

**Figure 6:**
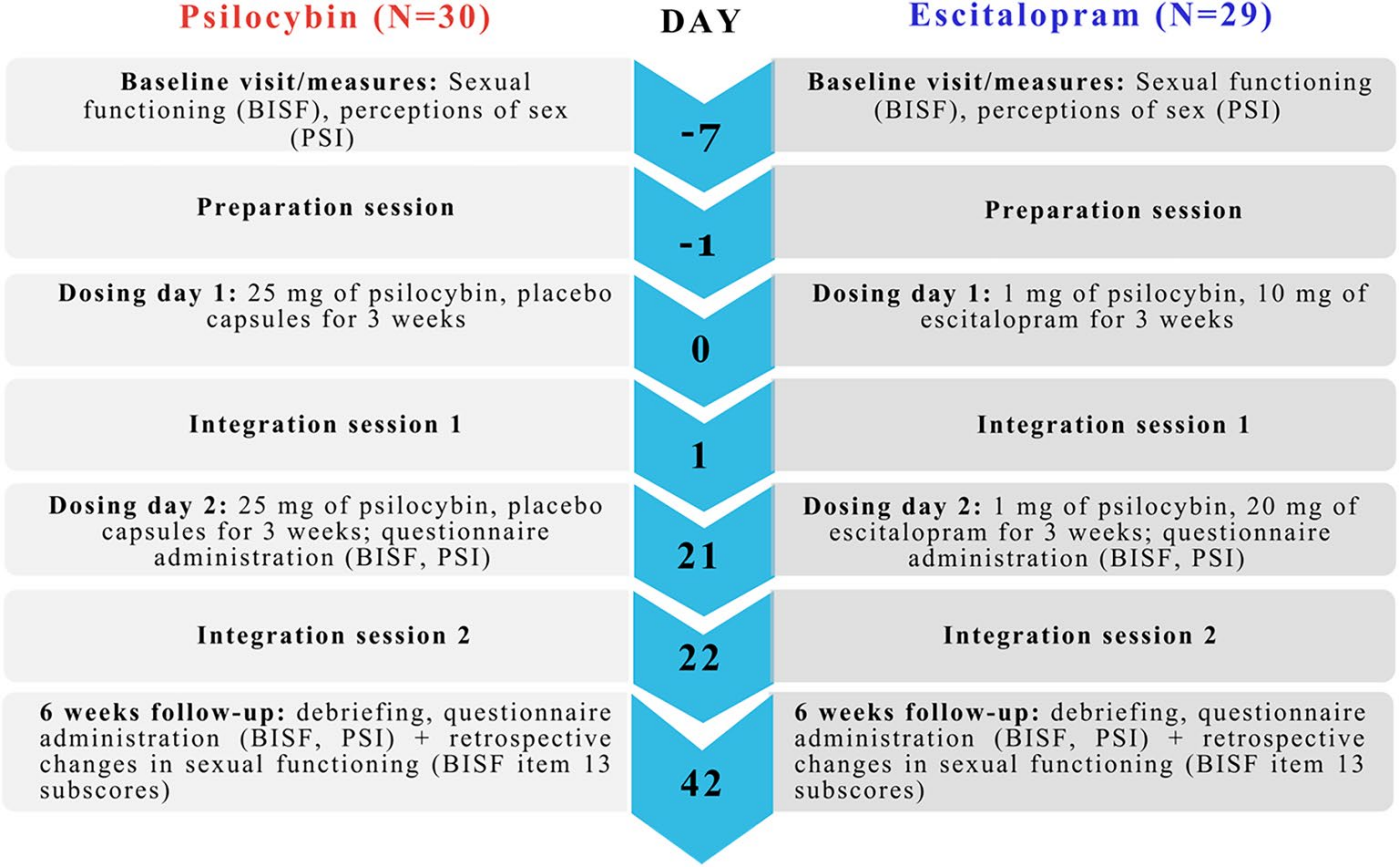
Overview of the DB-RCT trial procedure (Study 2). Numbers indicate days from baseline (day 0) to the 6-week trial primary end-point (day 42). The listed measures are only the ones included in the present study.

It comprises data derived from a phase II double-blind randomised controlled clinical trial (RCT) comparing psilocybin-therapy versus escitalopram treatment for major depression^7^. Participants had a diagnosis of moderate-severe MDD (> 17 on Hamilton-Depression [HAM-D-17^50^] scale at screening), were between 18 to 65 years old and were recruited through trial networks, social media, and other sources (see^7^ for demographic information). Participants were randomised to one of two arms: either receiving two doses of an active dose of psilocybin (25 mg) alongside 6 weeks of daily placebo (“psilocybin arm”), or two doses of a ‘control’ dose of psilocybin (1 mg) and daily escitalopram (10 mg for 3 weeks, then 20 mg for 3 weeks, “escitalopram arm”). During the active treatment period, each participant worked with two experienced therapists or psychiatrists administering an adapted form of Acceptance and Commitment Therapy^91^. On dosing days, the therapists accompanied them from the moment they ingested the drug until the day’s end. Before and after dosing days, participants underwent psychological preparation and integration, respectively. Taking into account screening, preparation, dosing, and integration, participants in each condition received approximately 20 h of in-person therapeutic support during the trial, as well as up to six further integration calls over Skype or by telephone. Licenses and approvals were obtained from the Home Office (Schedule 1), UK Medicines & Healthcare products Regulatory Agency (MHRA), Brent Research Ethics Committee (REC), the Health Research Authority (HRA) and Imperial College London (ICL) GDPR and the sponsors ICL Joint Research Compliance Office. Proprietary psilocybin was provided by COMPASS Pathways as ‘COMP360’ (Compass Pathways’ investigational, proprietary, synthetic, psilocybin formulation) and escitalopram by Guy’s and St Thomas’ Pharmacy. An overview of study 2 timepoints can be found in Fig. 6, see^7^ for further details on the study protocol and the main results of the trial. ClinicalTrials.gov Identifier: NCT03429075, registered on February 12, 2018; EudraCT: 2017-000219-18. CONSORT diagram for Study 2 can be found in Supplementary Materials.

### Measures

#### Sexual functioning and satisfaction

Consistent measures were used in Study 1 and 2. In Study 1, measures were employed at baseline (one week prior to the experience), 4 weeks, and 6 months after naturalistic psychedelic-use. In Study 2, measures were administered at baseline (1 week before dosing day 1) and at the 6-weeks follow-up, the RCT’s primary endpoint. Outcome measures were items extracted from the Brief Index of Sexual Functioning for Women (BISF-W), a standardized self-report measure of overall sexual function in women^86^. As the questionnaire was designed to be specifically used with women and our sample constituted of both men and women, we only used items that could be generalised to both sexes and we focused on the domains of experienced pleasure, communication, satisfaction of one’s partner, importance of sex, and satisfaction with one’s body image. We also did not use the full scale to limit the burden on participants, as a variety of other measures were also included. The questions and the response options were as follows: During the past month, have you felt pleasure from any forms of sexual experience? (0) I have not had a partner, (1) Have not felt any pleasure, (2) Seldom, less than 25% of the time, (3) Sometimes, about 50% of the time, (4) Usually, about 75% of the time, (5) Always felt pleasure. During the past month, how frequently have you been able to communicate your sexual desires or preferences to your partner/s?: (0) I have not had a partner/s, (1) I have been unable to communicate my desires or preferences, (2) Seldom, less than 25% of the time, (3) Sometimes, about 50% of the time, (4) usually, about 75% of the time, (5) I was always able to communicate my desires or my preferences. Overall, how satisfied have you been with your sexual relationship with your partner/s? (0) I have not had a partner/s, (1) Very satisfied, (2) Somewhat satisfied, (3) Neither satisfied nor dissatisfied, (4) Somewhat dissatisfied, (5) Very dissatisfied. Overall, how important is sexual activity in your life? (0) Not at all important, (1) Somewhat unimportant, (2) Neither important nor unimportant, (3) Somewhat important, (4) Very important. How satisfied you are with the overall appearance of your body? (0) Very satisfied, (1) Somewhat satisfied, (2) Neither satisfied nor dissatisfied, (3) Somewhat dissatisfied, (4) Very dissatisfied. If participants responded they did not have a partner in a question (response option 0), the answer was not included in the analysis for that specific item.

Additionally, we constructed two items to investigate whether psychedelics would be associated with a change in people’s (1) openness to sexual exploration and (2) perception of sex as a ‘spiritual experience’, where the latter term was not explicitly defined for the respondent. We conceived these 2 items after a review of the existing anecdotal reports of the effects of psychedelics on one’s sexual life^30–33^ and the cultural association between psychedelic-use, liberal sexual attitudes and behaviours and spiritual ideologies^2^. The items read as follows: “I am very open to trying out new things in my sex life” and “I see sex as a spiritual or sacred experience” and could be answered on a 7-point Likert scale.

Finally, exclusively in the 6 weeks follow-up of Study 2, we added item 13 from the BISF-W. This asks participants to retrospectively rate the level of change in any of the following areas of sexual functioning in the previous 6 weeks: (1) sexual interest, (2) sexual arousal, (3) sexual activity, (4) sexual satisfaction, (5) sexual anxiety. The response options were: (1) not applicable, (2) no change, (3) increase, (4) decrease.

#### Sexual dysfunction

To assess the appearance of sexual dysfunction after drug treatment in Study 2 we used the Psychotropic-Related Sexual Dysfunction Questionnaire (PRSexDQ-SALSEX^94^). The scales include 7 items assessing SD. The first is a screening item that assesses if the patient experienced any sort of SD during treatment. The second item assesses whether the patient has spontaneously reported any SD to his or her physician. The next items (items 3–7) assess five dimensions of SD according to severity or frequency: loss of libido (0 = nil, 1 = mild, 2 = moderate, 3 = severe), delayed orgasm or ejaculation (0 = nil, 1 = mild, 2 = moderate, 3 = severe), lack of orgasm or ejaculation (0 = never, 1 = occasionally, 2 = often, 3 = always), erectile dysfunction in men/vaginal lubrication dysfunction in women (0 = never, 1 = occasionally, 2 = often, 3 = always), and patient's tolerance of the SD (0 = no sexual dysfunction, 1 = good, 2 = fair, 3 = poor). Only items 3 through 7 account for the total score of the PRSexDQ-SALSEX. Sexual dysfunction is scored as mild = 1–5 (with no item > 1); moderate = 6–10 (OR any item = 2, with no item = 3) or severe = 11–15 (OR any item = 3). As the scale is designed for retrospective use, it was only collected at the 6-weeks follow-up of the trial.

#### Well-being

The Flourishing Scale^95^ is a brief 8-item summary measure of the respondent’s self-perceived success in important areas such as relationships, self-esteem, purpose, and optimism. The scale provides a single psychological well-being score. The scores range from 8 to 56. A high score represents a person with many psychological resources and strengths.

#### Depression

Depressive symptoms were assessed with the 16-item Quick Inventory of Depressive Symptomatology Self-Report^96^. The total score establishes the severity of depression, ranging from ‘absent’ (0–5) to ‘mild’ (6–10), ‘moderate’ (11–15), ‘severe’ (16–20) and ‘very severe’ (21–27).

### Statistical analyses

#### Study 1

Changes on the individual items of the adapted BISF-W from baseline to 4 weeks and 6 months after the psychedelic experience were assessed via non-parametric Friedman rank sum tests due to the ordinal nature of the response items. Wilcoxon signed-rank tests between baseline, 4-week, and 6-month endpoints were used as follow-up tests. Additionally, spearman correlations between changes on individual items of the BISF-W and changes in flourishing (FS) from baseline to the 4-week endpoint are reported in order to investigate if changes in sexual functioning correlated with changes in wellbeing. Finally, cumulative links models were fitted in order to investigate differences between male and female participants on any of the sexuality-related items (Supplementary Material 1).

#### Study 2

Due to the limited sample size and structure of the Likert-item based data, cumulative link models for ordinal regression were performed to compare changes in BISF-W items between the psilocybin and escitalopram arms of the RCT^97^. Cumulative link models are structurally related to mixed linear models, in that they allow fitting random intercepts and slopes on ordinal, instead of continuous data. For the present sample, models with random intercept only were found to produce the best fit indices, based on Bayesian Information Criteria (BIC). Symmetric threshold parameters were chosen for items rated from Strongly Disagree to Strongly Agree, while equidistant thresholds were used for items rated using equally spaced numerically defined proportions (e.g., None of the time, 25% of the time, 50% of the time, etc.). Post-hoc within-group contrasts were calculated based on estimated marginal means for all items. Rosenthal correlation coefficients (R) were added as effect size (EF) estimates in Table 3. They are calculated by dividing the z value by the sqrt of the sample size^98^. These coefficients are commonly used in the case of ordinal variables and a value of 0.00 < 0.20 indicates a very low ES, 0.20 < 0.40 low ES, 0.40 < 0.60 moderate ES, 0.60 < 0.80 strong ES, 0.80 < 1.00 very strong ES. Scores on the BISF-W item 13, which was only included at the endpoint, were compared between the groups via Mann Whitney U tests, where rank-biserial correlation coefficients (r) ≥ 0.3 was defined as a small, r ≤ 0.5 medium and r > 0.5 as a large effect. Ordinal scores from the PRSexDQ-SALSEX, which was also only included at the endpoint, were also compared using a Mann Whitney U test in order to investigate differences in the severity of sexual dysfunction between the two groups.

Due to the small sample size, Bonferroni-corrected spearman correlations between longitudinal changes in depressive symptoms (QIDS-SR-16) and SF were calculated based only on the retrospective BISF-W item 13 investigating retrospective changes in sexual interest, arousal, activity, satisfaction, and anxiety in order avoid inflation of the number of tests. These correlations investigated if changes in depression correlated with changes in sexual functioning.

### Supplementary Information

Supplementary Information.

## Data Availability

The data that support the findings of this study are available from the corresponding author, [TB], upon reasonable request.
