# Supplementary material for: Psychedelics and sexual functioning: a mixed-methods study
Source: Sci Rep. 2024 Feb 7;14:2181. doi: 10.1038/s41598-023-49817-4 (PMC10850066; doi:10.1038/s41598-023-49817-4)
Supplement: Supplementary file 1 — Supplementary Information. [file 41598_2023_49817_MOESM1_ESM.docx]

**Supplementary Materials**

**Supplementary Materials 1: Consort Diagrams**

1. **Study 1 (survey study)**

## Enrollment

Baseline completers

(n= 1463)

Analysed

(n= 261)

## Analysis

Excluded for not completing baseline of 4 weeks

(n= 61)

6-months follow-up

(n= 322)

4-weeks primary endpoint

(n= 718)

## Follow-Up

1. **Study 2 (Randomized controlled trial)**

**
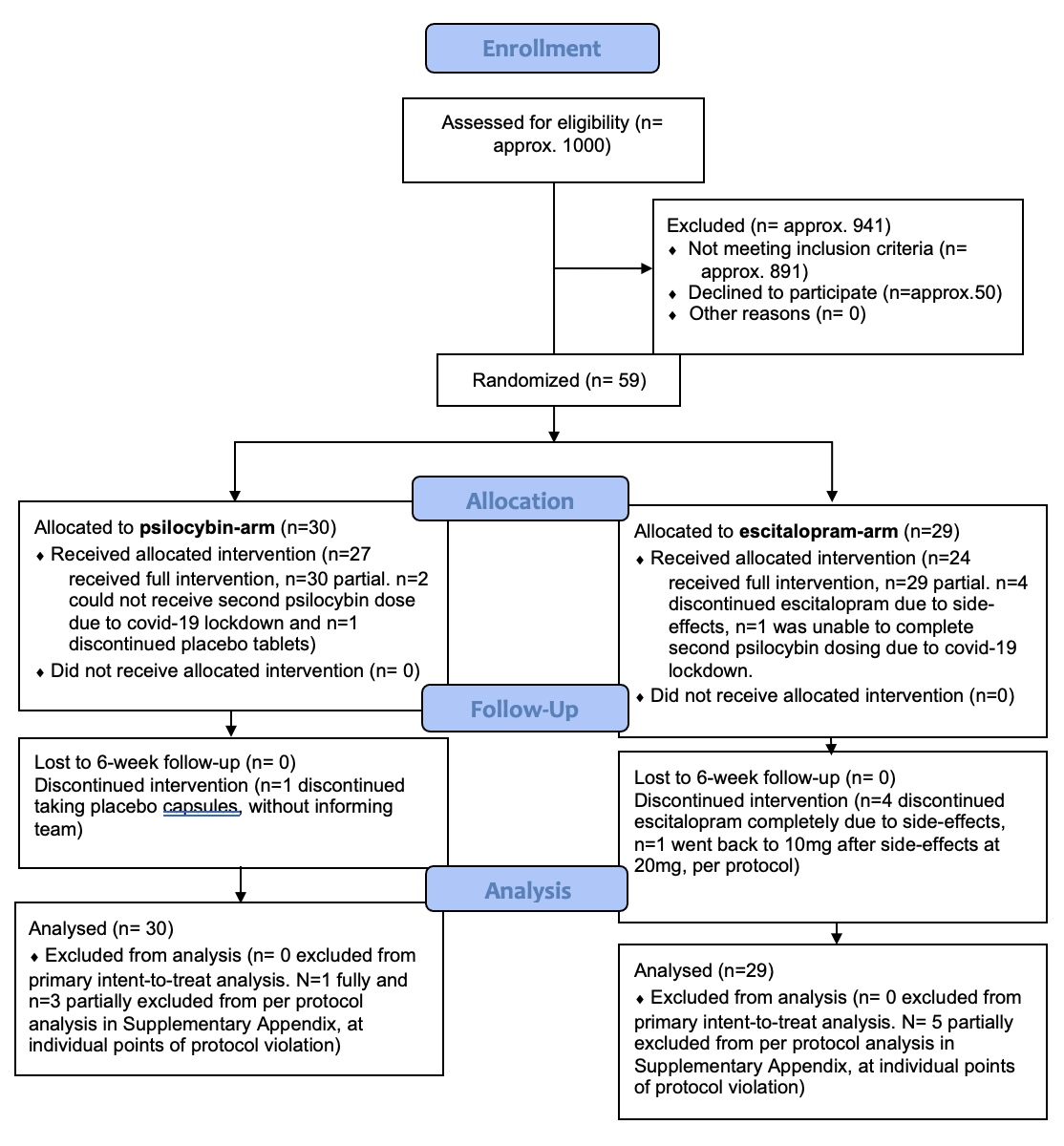
**

**Supplementary Materials 2: Gender Analyses**

1. **Results**

Cumulative links model were fitted in order to investigate differences between male and female participants on any of the sexuality-related items. Type II analyses of deviance (ANOVAs) were performed to assess main and interaction effects of time and gender on all included items included in the main analysis. No significant interaction effects were detected for any of the items (p> .05), except for ‘partner satisfaction’ (LR Chisq = 7.73, p = .02). Inspection of coefficients showed that this interaction was driven by a reduction in ratings at the final 6-month endpoint for female, compared to male participants (beta = 1.17, p < .01), descriptively showing a return of partner satisfaction levels back to baseline in female but not male participants. Main effects of time were in line with results of the main analysis, showing significant changes across genders for all items other than importance, with the greatest changes present for the ‘sex as spiritual’ item (LR Chisq = 39.56, p < .0001). Main effects of gender were detected for all items, with male participants giving higher ratings on their perceived pleasure (LR Chisq= 19.26, p < .0001), appearance satisfaction  (LR Chisq= 13.30, p < .01), sexual openness  (LR Chisq= 20.56, < .0001), and most strongly, importance of sex  (LR Chisq= 18.75, p < .01), while females showed higher ratings on their ability to communicate sexual desires and preferences  (LR Chisq=7.19, p <.01), and seeing sex as spiritual  (LR Chisq= 17.95, p< .001) across time points. More information can be found in Supplementary Figure 1.

**Supplementary Figure 1.** Single item analyses assessing changes in sexual functioning and satisfaction in both males and females after naturalistic psychedelic use in a sample of N = 261 completers at 4 weeks and 6-month follow-up. ‘n.s’ indicates that the difference between baseline and follow-up scores is non-significant (P > 0.05). ***The difference between baseline and follow-up scores is significant, with a P < .0001. **The difference between baseline and follow-up scores is significant, with a P < .001. *The difference between baseline and follow-up scores is significant, with a P < .01. Error bars represent SE(M).
